# Supplementary material for: R-Spondin 2 governs Xenopus left-right body axis formation by establishing an FGF signaling gradient
Source: Nat Commun. 2024 Feb 2;15:1003. doi: 10.1038/s41467-024-44951-7 (PMC10837206; doi:10.1038/s41467-024-44951-7)

Supplementary Information

R-Spondin 2 governs *Xenopus* left-right body axis formation by establishing an FGF signaling gradient

Hyeyoon Lee[1], Celine Marie Camuto[1] and Christof Niehrs[1,2*]

[1]Division of Molecular Embryology, DKFZ-ZMBH Alliance, Deutsches Krebsforschungszentrum (DKFZ), 69120 Heidelberg, Germany
[2]Institute of Molecular Biology (IMB), 55128 Mainz, Germany

*Correspondence: niehrs@dkfz-heidelberg.de

Supplementary Figures 1-8
Uncropped scan of blots from Supplementary Figures

Supplementary Figure 1

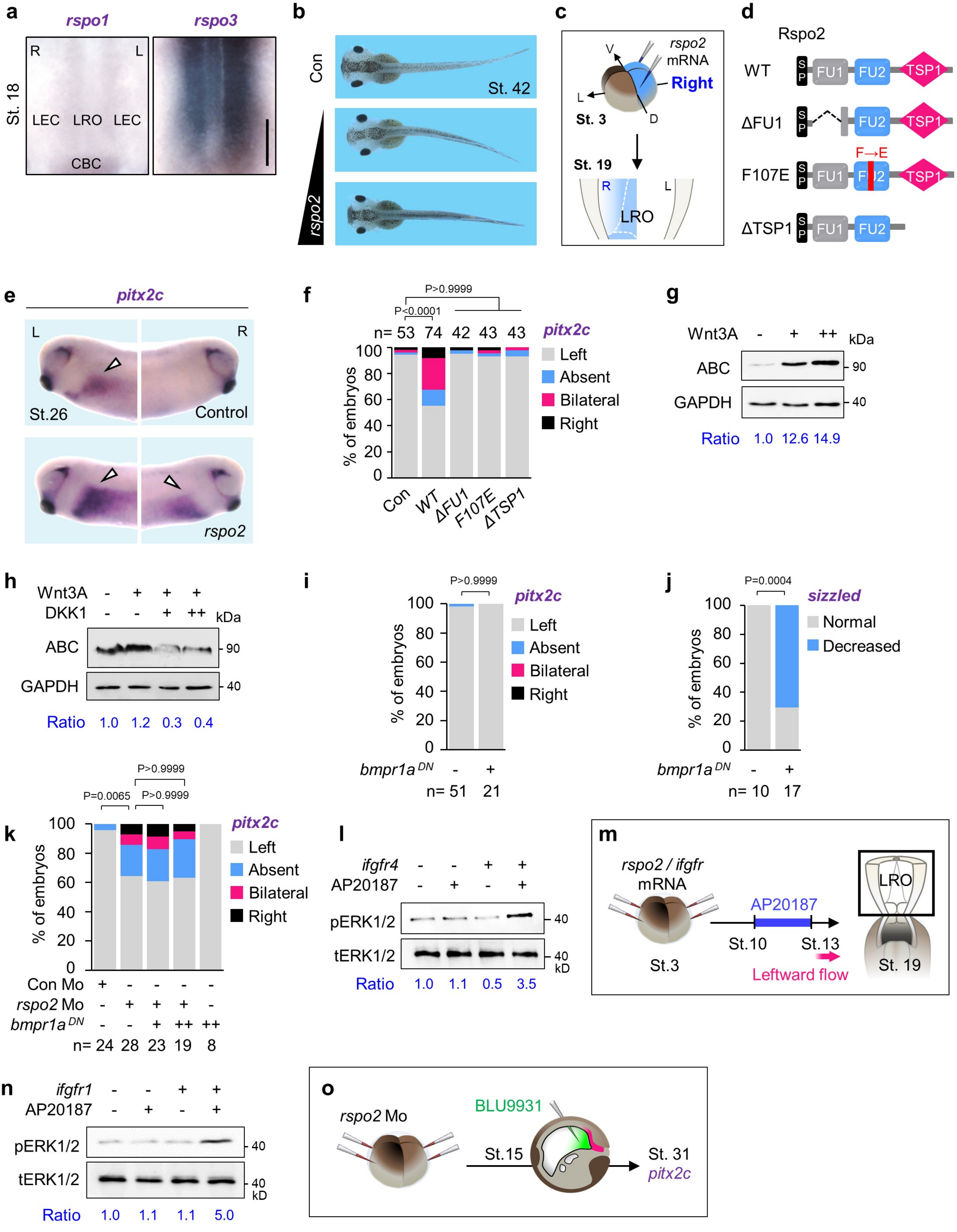

Supplementary Figure 1. Rspo2 regulates LR asymmetry Wnt and BMP independently.

(**a**) Whole-mount *in situ* hybridization (WISH) of *rspo1* and *rspo3* in the LRO (St. 18). LEC, lateral-endodermal crest; CBC, circumblastoporal collar. Scale bar, 100 µm.

(**b**) Representative images of *Xenopus* tailbuds (St. 42) in **Fig. 1**. Note that the *rspo2* mRNA dose used was below that causing gastrulation defects and embryos developed with normal body axis.

(**c**) Microinjection strategy for (**d-f**).

(**d**) Domain structures of Rspo2 wildtype (WT) and Rspo2 mutants. SP, signal peptide; FU, furin domain; TSP1, thrombospondin domain 1.

(**e**) WISH of *pitx2c* in *Xenopus* tailbuds (St. 26) injected as indicated. Left (L) and right (R) sides of the same embryos are shown. Arrowheads, *pitx2c* at the LPM. Scale bar, 0.5 mm.

(**f**) Quantification of *pitx2c* from Rspo2 and Rspo2 mutants injected embryos.

(**g-h**) Western blot analyses in *Xenopus* neurulae to validate Wnt3A and DKK1 activities in Wnt signaling. Ratio, relative levels of activated β-catenin (ABC) normalized to GAPDH.

(**i**) Quantification of *pix2c* WISH in *Xenopus* tailbuds (St. 31) injected with *bmpr1a*^{DN} mRNA.

(**j**) Quantification of *sizzled* WISH in *Xenopus* gastrulae (St. 11) injected with *bmpr1a*^{DN} mRNA.

(**k**) Quantification of *pitx2* WISH in *Xenopus* tailbuds (St. 31) injected as indicated.

(**l**) Western blot analysis of phosphorylated ERK1/2 (pERK1/2) and total ERK1/2 (tERK1/2) with *Xenopus* neurulae (St. 19) lysates injected and treated as indicated. Ratio, relative levels of pERK1/2 normalized to tERK1/2.

(**m**) Microinjection strategy for **Figure 3j**-**k**.

(**n**) Western blot analysis of phosphorylated ERK1/2 (pERK1/2) and total ERK1/2 (tERK1/2) with *Xenopus* neurulae (St. 19) lysates injected and treated as indicated. Ratio, relative levels of pERK1/2 normalized to tERK1/2.

(**o**) Microinjection strategy for **Figure 3l**.

Data information: Two-sided Fisher's exact test used for all statistical analyses. n=number of embryos. Source data are provided as a Source Data file.

Supplementary Figure 2

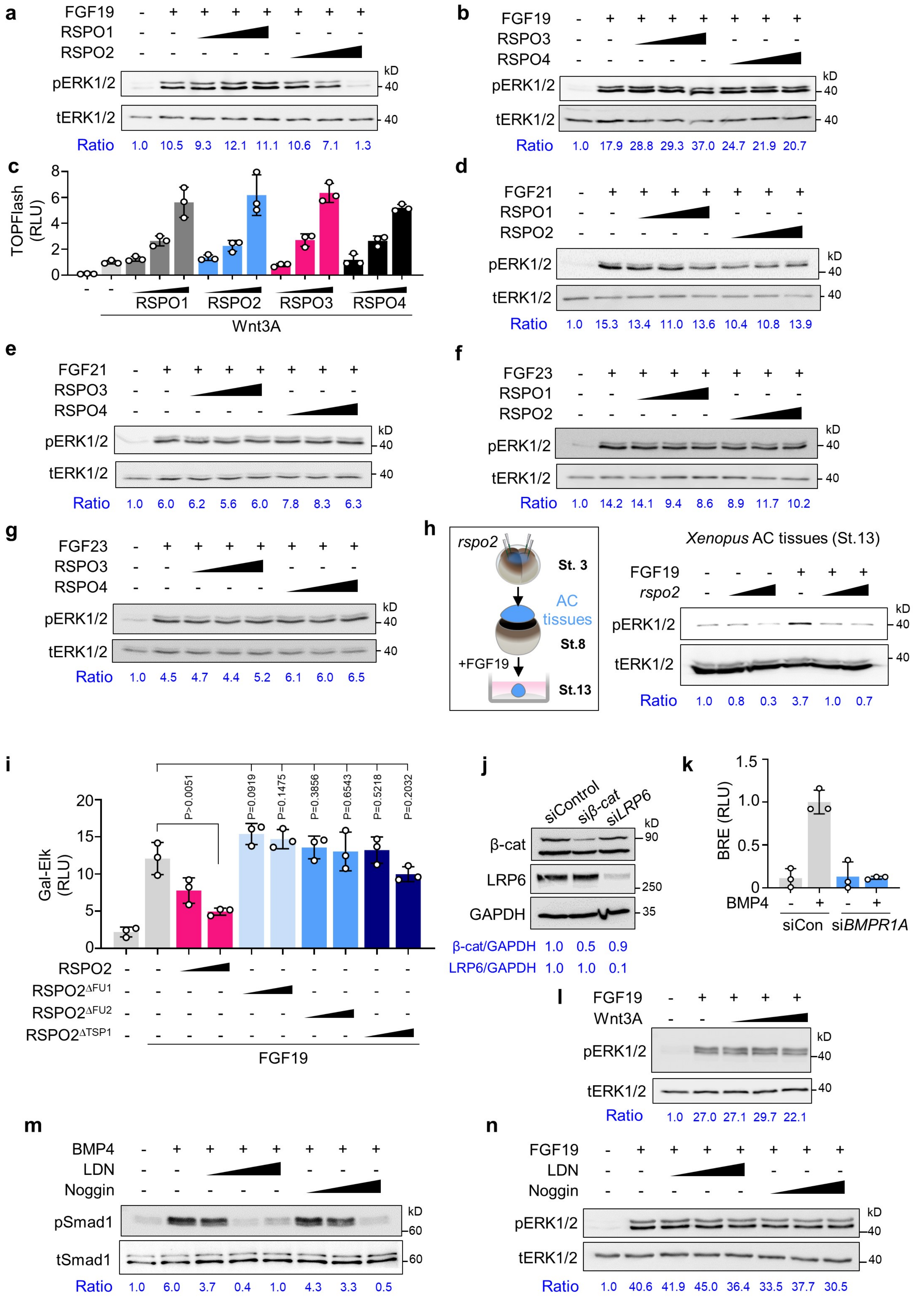

Supplementary Figure 2. RSPO2 antagonizes FGF19-FGFR4 signaling.

(**a-b**) Western blot analyses of phosphorylated ERK1/2 (pERK1/2) and total ERK1/2 (tERK1/2) in HEPG2 cells stimulated by FGF19, treated with or without increasing amount of RSPOs for 30 min as indicated.

(**c**) TOPFlash reporter assay in HEK293T cells treated with Wnt3A and RSPOs as indicated.

(**d-g**) Western blot analyses of pERK1/2 and tERK1/2 in HEPG2 cells stimulated by FGF21 or FGF23, treated with or without increasing amounts of RSPOs for 30 min as indicated.

(**h**) (Left) Scheme of analysis. (Right) Western blot analysis of pERK1/2 and tERK1/2 in Xenopus animal cap explants treated with FGF19 from St. 8 to St. 13.

(**i**) FGF-responsive GAL-Elk1 luciferase reporter assay in HEPG2 cells treated with FGF19 and RSPOs as indicated.

(**j**) Western blot analysis in HEPG2 cells upon siRNA transfection.

(**k**) BMP-responsive BRE reporter assay in HEPG2 cells treated as indicated.

(**l-n**) Western blot analyses in HEPG2 cells treated as indicated.

Data information: For **a, b, d, e, f, g, h, l** and **n**: Ratio, relative levels of pERK1/2 normalized to tERK1/2. For all reporter assays, data are displayed as mean ± SD with two-tailed unpaired t-test. n=3 biologically independent samples. Source data are provided as a Source Data file.

Supplementary Figure 3

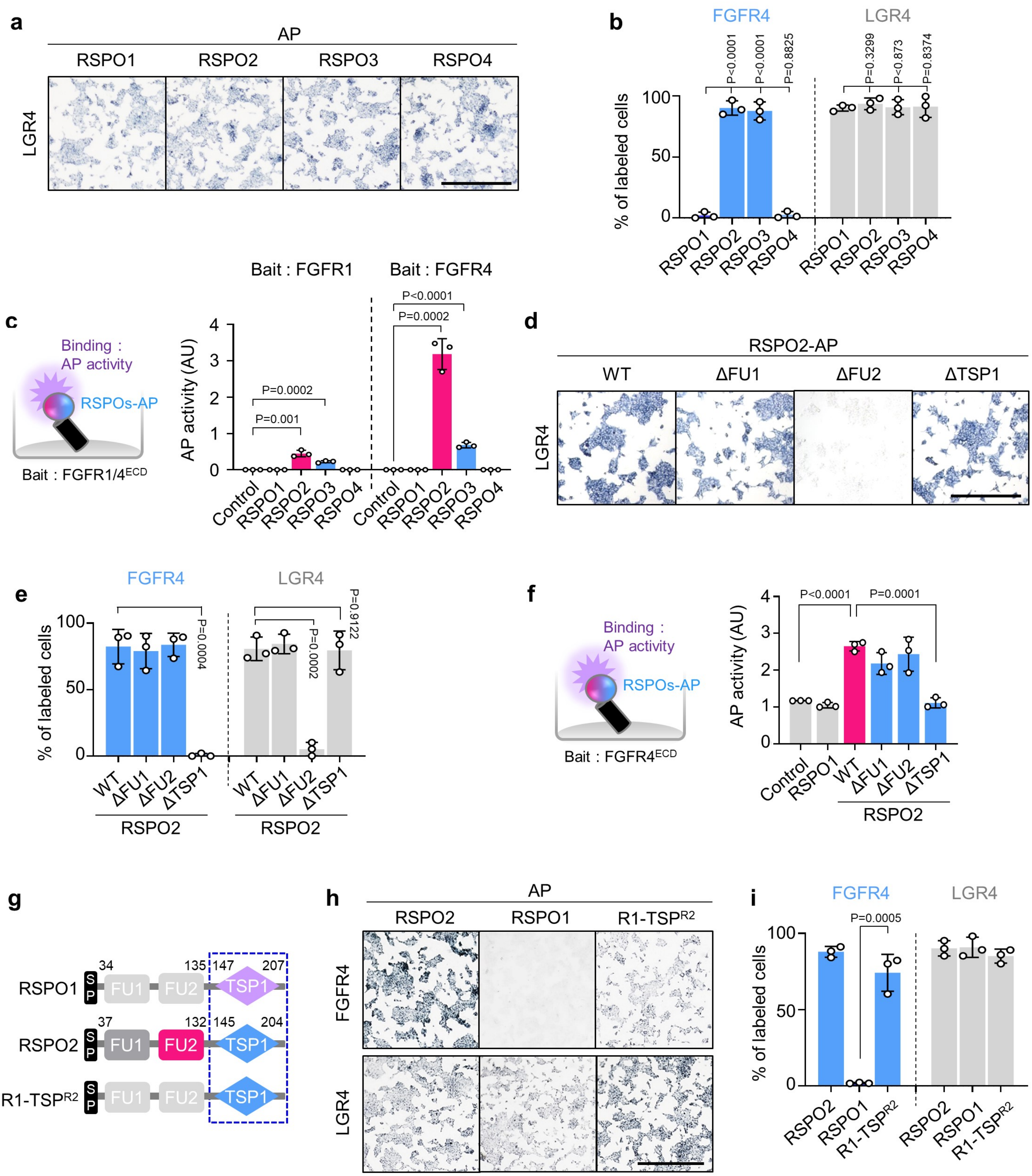

Supplementary Figure 3. RSPO2 interacts with FGFR4 via the TSP1 domain.

(**a**) RSPOs cell surface binding assays in HEK293T cells transfected with LGR4 and treated with RSPOs as indicated. Scale bar, 1 mm.

(**b**) Quantification of **Figure 6b** and (**a**). n=3 biologically independent samples. Data are displayed as means ± SD with two-tailed unpaired t-test.

(**c**) (Left) Scheme of analysis. (Right) *In vitro* binding assay between RSPOs and FGFR1/4. n=3 experimentally independent samples. Data are displayed as mean ± SD with two-tailed unpaired t-test,

(**d**) Cell surface binding assay in HEK293T cells transfected with LGR4 and treated with RSPOs as indicated. Scale bar, 1 mm.

(**e**) Quantification of **Figure 6e** and (**d**). n=3 biologically independent samples. Data are displayed as means ± SD with two-tailed unpaired t-test.

(**f**) (Left) Scheme of analysis. (Right) *In vitro* binding assay between RSPOs and FGFR4. n=3 experimentally independent samples. Data are displayed as means ± SD with two-tailed unpaired t-test.

(**g**) Domain structures of the RSPO1, -2 and R1-TSP^{R2}. Dashed box denotes the TSP1 domain swapping.

(**h**) Cell surface binding assay in HEK293T cells. Scale bar, 1 mm.

(**i**) Quantification of (**h**). n=3 biologically independent samples.

Data information: Data are displayed as means ± SD with two-tailed unpaired t-test. Source data are provided as a Source Data file.

Supplementary Figure 4

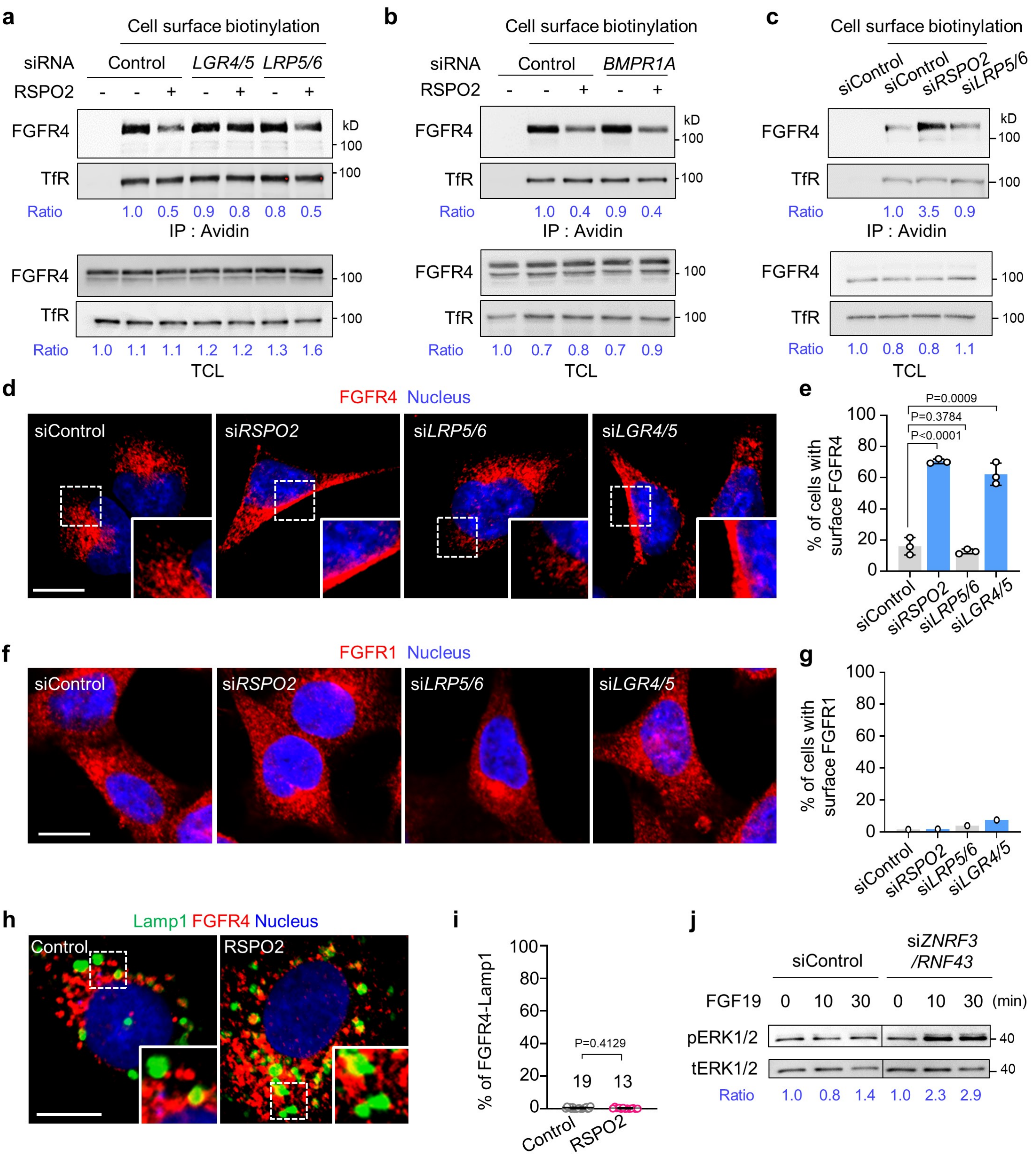

Supplementary Figure 4. RSPO2 removes cell surface FGFR4.

(**a**-**b**) Cell surface biotinylation assays in HEPG2 cells treated as indicated. Transferrin receptor (TfR), a loading control. TCL, Total cell lysate. Ratio, relative levels of cell surface FGFR4 normalized to cell surface TfR.

(**c**) Cell surface biotinylation assay in H1581 cells upon siRNA transfection as indicated. Ratio, relative levels of cell surface FGFR4 normalized to cell surface TfR.

(**d**) IF of endogenous FGFR4 in H1581 cells upon siRNA transfection as indicated. Nuclei were stained with Hoechst. Scale bar, 20 µm.

(**e**) Quantification of cells harboring surface FGFR4 from (**e**). Data are displayed as mean ± SD with two-tailed unpaired t-test. n= 3 biologically independent experiments.

(**f**) IF of endogenous FGFR1 in H1581 cells upon siRNA transfection as indicated. Nuclei were stained with Hoechst. Scale bar, 20 µm. n= single experiment.

(**g**) Quantification of cells harboring surface FGFR1 from (**f**).

(**h**) Co-IF of Lamp1-FGFR4 in HEPG2 cells upon RSPO2 treatment. Nuclei were stained with Hoechst. Scale bar, 20 µm.

(**i**) Quantification of (**h**). Data are displayed as mean ± SD with two-tailed unpaired t-test. n= number of cells.

(**j**) Western blot analysis in H1581 cells treated with siRNA and stimulated by FGF19 as indicated. Ratio, relative levels of pERK1/2 normalized to tERK1/2. Source data are provided as a Source Data file.

Supplementary Figure 5

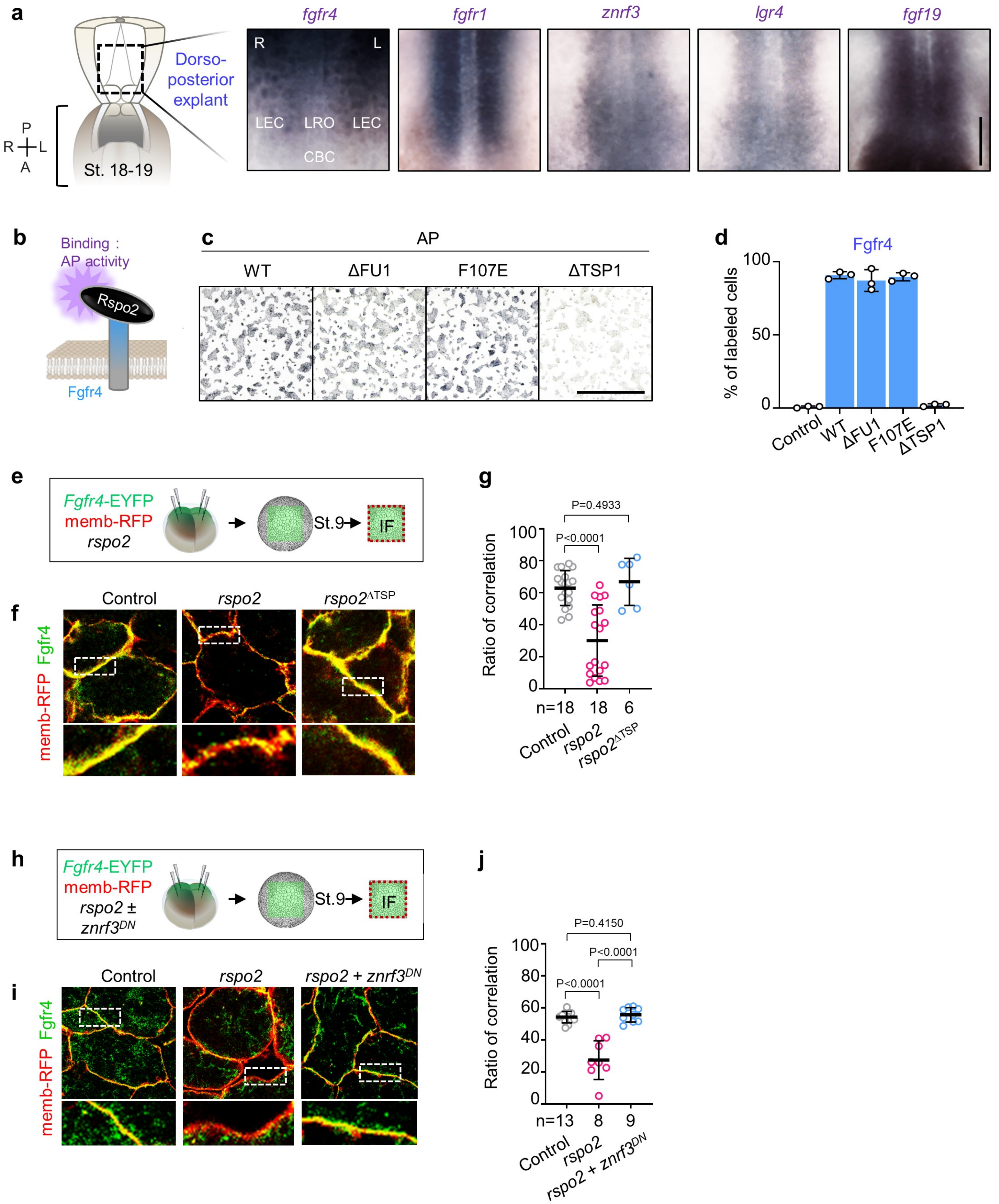

Supplementary Figure 5. Rspo2 removes cell surface Fgfr4 in *Xenopus* embryo.

(**a**) Whole-mount *in situ* hybridization (WISH) of *fgfr4, fgfr1, znrf3, lgr4,* and *fgf19* in *Xenopus* LRO (St. 18-19). R, right; L, left. LEC, lateral-endodermal crest; CBC, circumblastoporal collar. Scale bar, 100 µm.

(**b**) Scheme of cell surface binding assay in (**c-d**).

(**c**) Cell surface binding assay in HEK293T cells transfected with *Xenopus* Fgfr4 and treated with *Xenopus* Rspo2 wildtype and Rspo2 mutants as indicated. Scale bar, 1 mm.

(**d**) Quantification of (**c**). n=3 biologically independent samples. Data are displayed as means ± SD.

(**e**) Scheme for immunofluorescence microscopy (IF) in *Xenopus* animal cap (AC) explants in (**f**). Embryos were injected animally at St. 3 with *fgfr4*-EYFP and membrane (memb)-RFP mRNA along with wild-type or the TSP1 deletion mutant *rspo2* mRNA. AC explants were dissected at St.9 for IF. Memb-RFP was served as a control to compare reduction of Fgfr4-EYFP level at cell surface.

(**f**) IF for Fgfr4 (green) and cell surface (red) in AC explants injected as indicated, with a representative cell (top) and magnification (inset). Scale bar, 20 µm.

(**g**) Quantification of (**f**). n= number of areas analyzed. Data are displayed as mean ± SD with two-tailed unpaired t-test.

(**h**) Scheme for IF in *Xenopus* AC explants in (**i**). Embryos were injected animally at St. 3 as indicated and dissected at St.9 for IF.

(**i**) IF for Fgfr4 (green) and cell surface (red) in AC explants injected as indicated, with a representative cell (top) and magnification (inset). Scale bar, 20 µm.

(**j**) Quantification of (**i**). n= number of areas analyzed.

Data information: Data are displayed as mean ± SD with two-tailed unpaired t-test. Source data are provided as a Source Data file.

Supplementary Figure 6

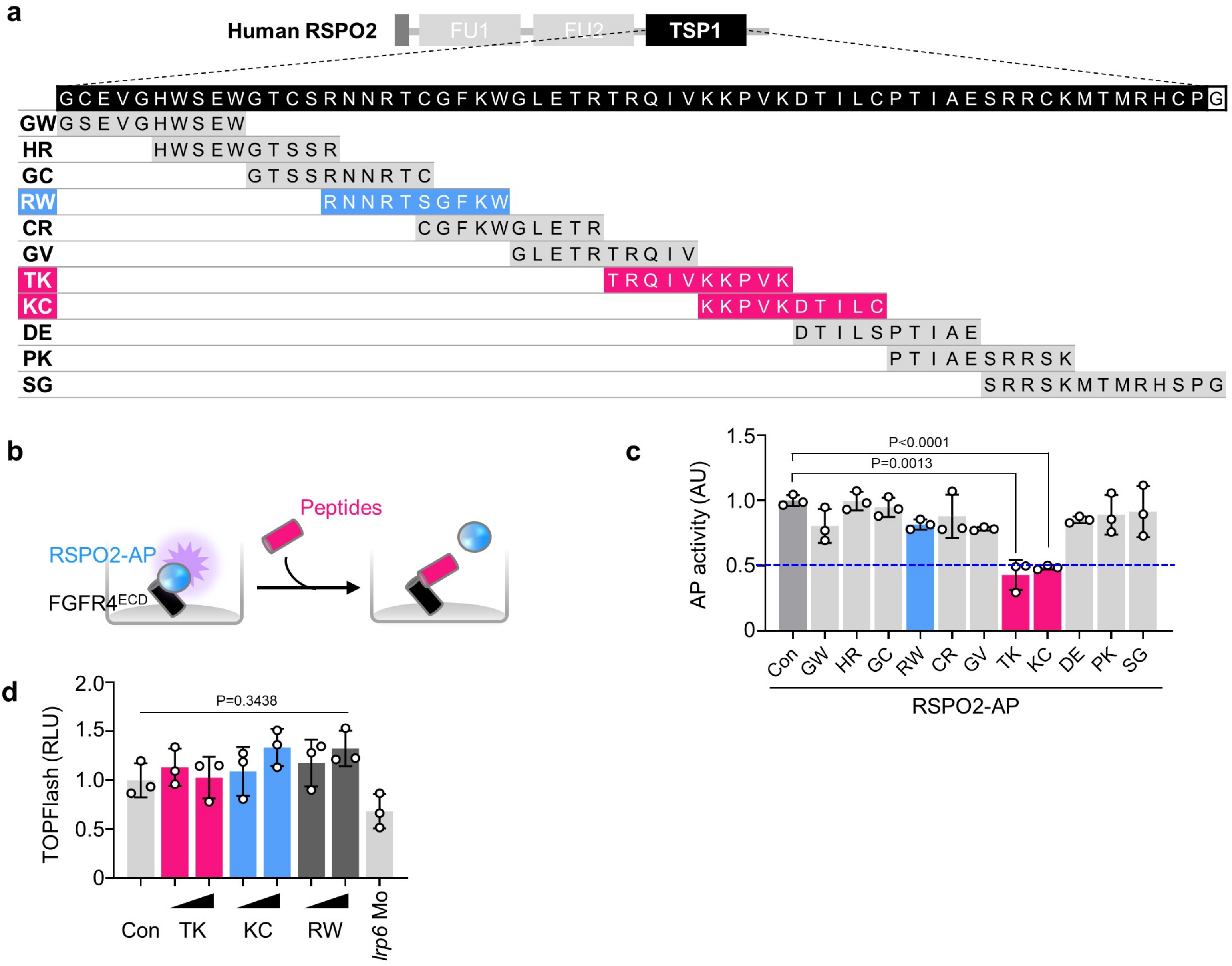

Supplementary Figure 6. Identification of RSPO2-FGFR4 intervening peptides TK/KC.

(**a**) Amino acid sequence (aa 142-206) of human RSPO2 TSP1 domain and overlapping peptide candidates derived from the TSP1 domain. 11 peptides harboring 10-14 amino acids were designed with 5 amino-acids overlapping. Cysteine residues in the middle of each peptide candidate were substituted by serine residues.

(**b**) Scheme for *in vitro* competitive binding assay in (**c**). $FGFR4^{ECD}$ was coated on plastic wells as a bait, followed by RSPO2-AP treatment for 3 hours with or without 100 μM of peptide candidates. Binding between RSPO2 and $FGFR4^{ECD}$ was detected with AP activity.

(**c**) *In vitro* binding assay for RSPO2 and $FGFR4^{ECD}$ interaction competing with overlapping peptides. Data are displayed as means ± SD with two-tailed unpaired t-test. n= 3 experimental replicates.

(**d**) TOPFlash reporter assay in *Xenopus* neurula (St. 15) injected with peptides.

Data information: Data are displayed as means ± SD with ordinary one-way ANOVA test. n=3 biologically independent samples. Note that no peptide inhibits Wnt signaling. Source data are provided as a Source Data file.

Supplementary Figure 7

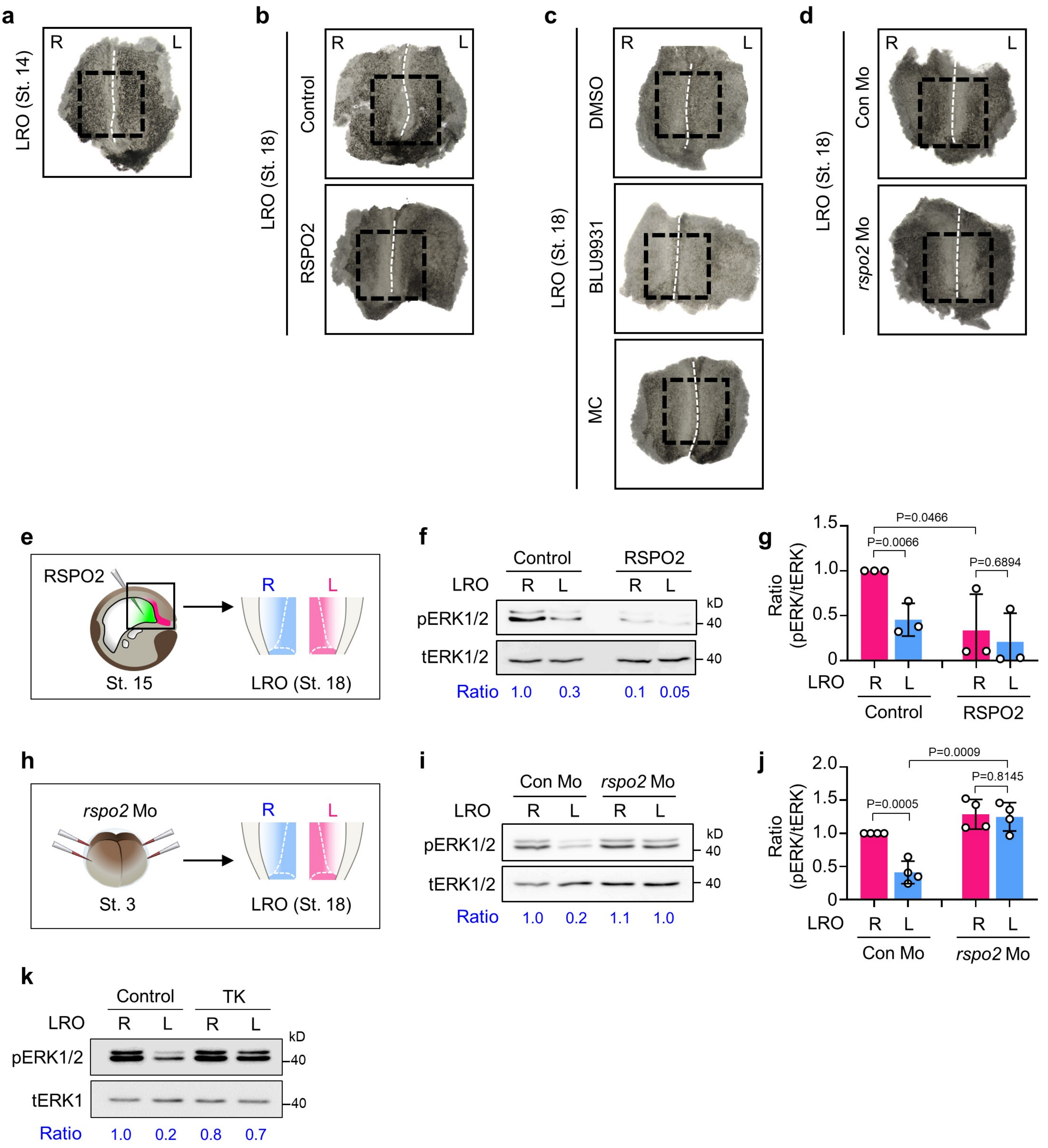

Supplementary Figure 7. Rspo2 is required for LR asymmetric ERK1/2 phosphorylation in the LRO.

(**a-d**) Brightfield image of the LRO explants immunostained with phosphorylated ERK1 (pERK1) shown in **Figure 7**. R, right; L, left. White dashed line, midline; Black dashed box, area of IF shown in **Figure 7**.

(**e**) Scheme of gastrocoel injection and the LRO dissection in (**f, g**).

(**f**) Representative western blot analyses of right (R) and left (L) side LROs dissected from *Xenopus* St. 18 neurula injected as indicated.

(**g**) Quantification of western blot analyses described in (**f**). n=3 biologically independent samples. Data show relative levels of pERK1/2 normalized to tERK1/2 as means ±SEM. Normalized pERK/tERK level in the right-side LRO from control embryo was set to 1.

(**h**) Scheme of microinjection and the LRO dissection in (**i, j**).

(**i**) Representative data from western blot analyses in the right (R) and the left (L) side LROs.

(**j**) Quantification of western blot analyses described in (**i**). n=4 biologically independent samples.

(**k**) Western blot analyses in the right (R) and the left (L) side LROs dissected from *Xenopus* St. 18 neurula injected as indicated.

Data information: Ratio, relative levels of pERK1/2 normalized to tERK1/2. Two-tailed unpaired t-test was used for all statistical analyses. Source data are provided as a Source Data file.

Supplementary Figure 8

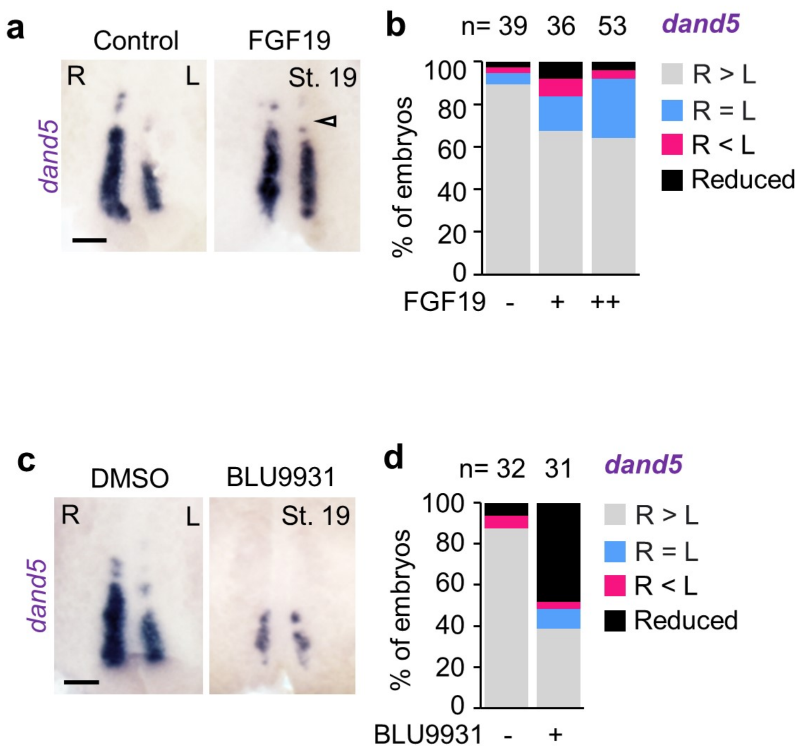

Supplementary Figure 8. FGF signaling increases *dand5* expression in the LRO.

(**a**) WISH of *dand5* in *Xenopus* LRO (St. 19) injected as indicated. Arrowhead, derepression of *dand5*. Scale bar, 100 µm.

(**b**) Quantification of (**a**). n= number of dorso-posterior explants.

(**c**) WISH of *dand5* in *Xenopus* LRO (St. 19) injected as indicated. Scale bar, 100 µm.

(**d**) Quantification of (**c**). n= number of dorso-posterior explants.

Source data are provided as a Source Data file.

Uncropped scans of blots from Supplementary Figure 1

g

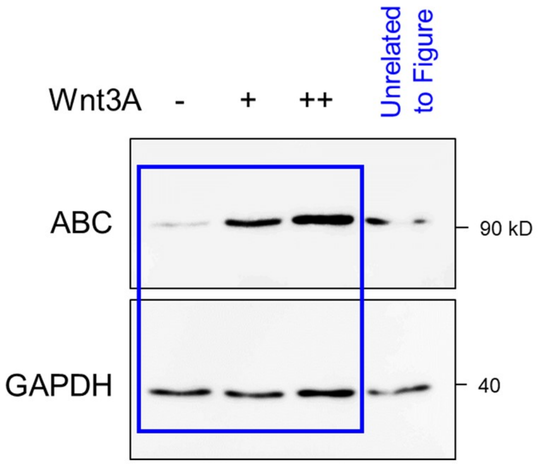

h

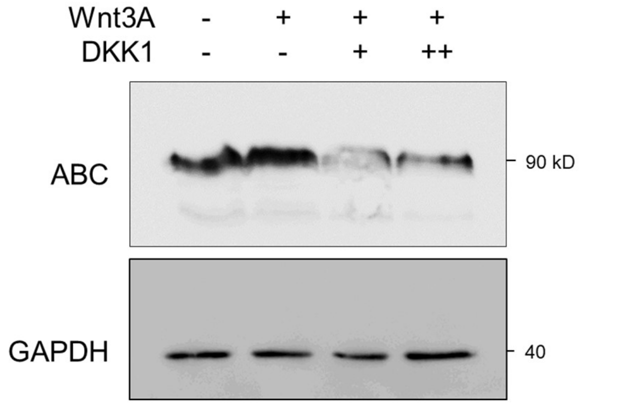

l

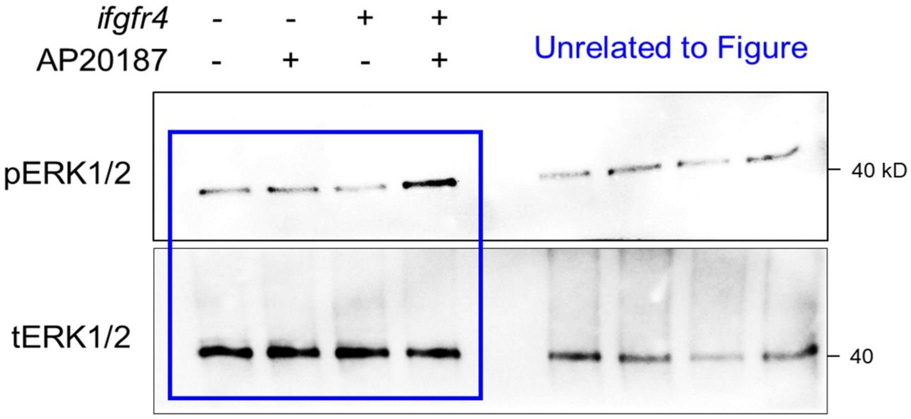

n

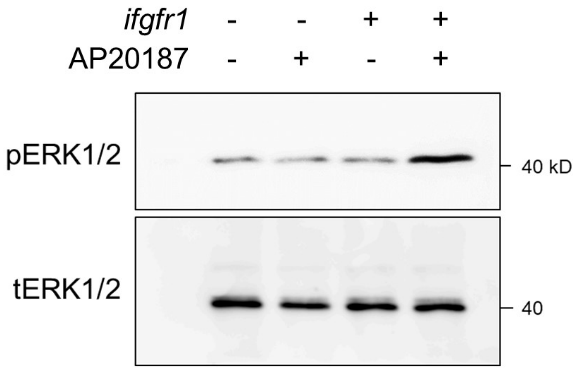

Uncropped scans of blots from Supplementary Figure 2

a

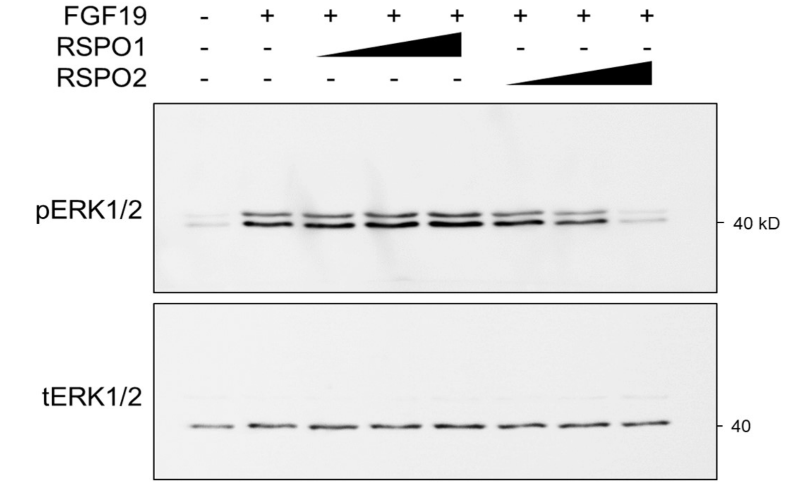

b

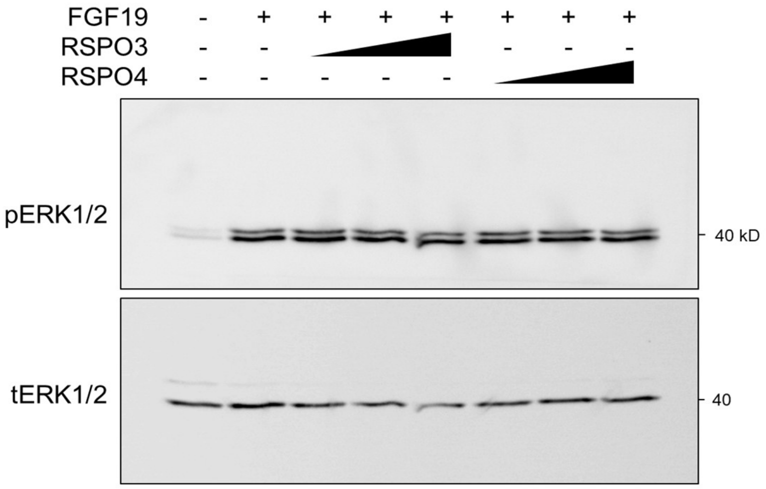

d

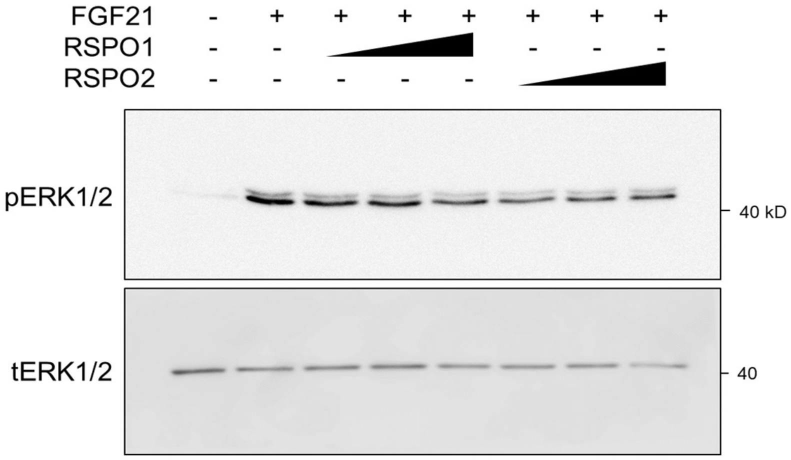

e

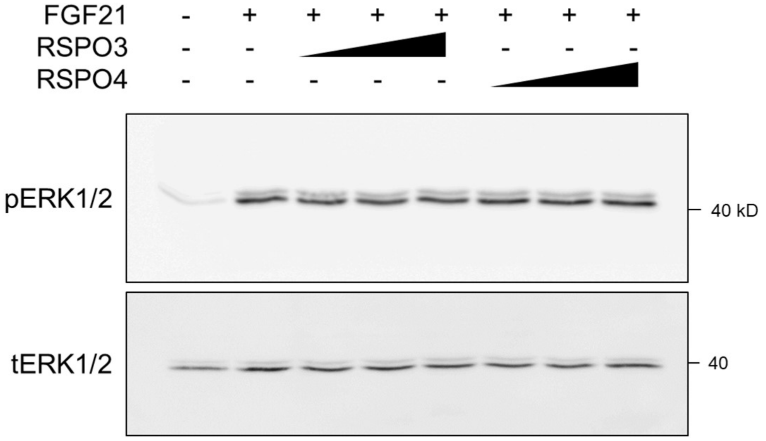

f

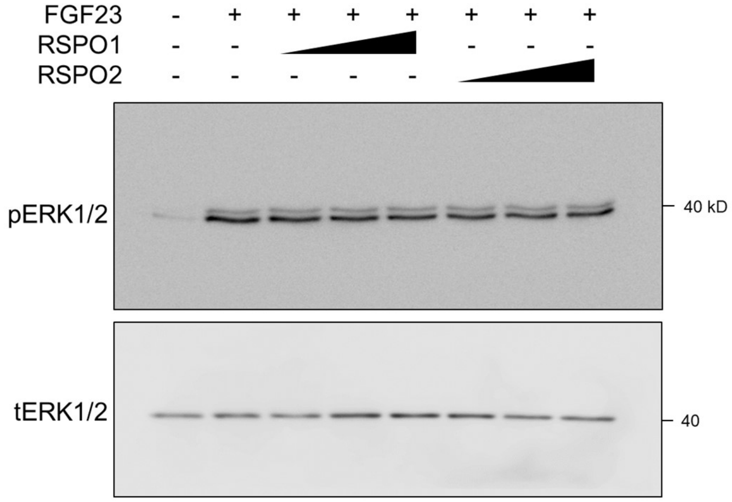

g

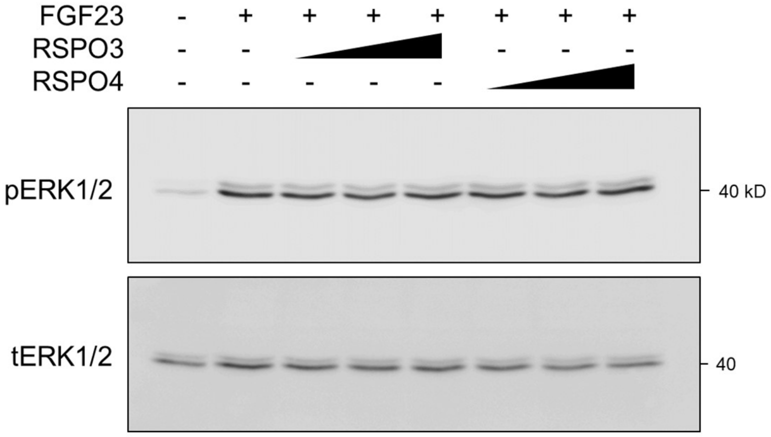

Uncropped scans of blots from Supplementary Figure 2

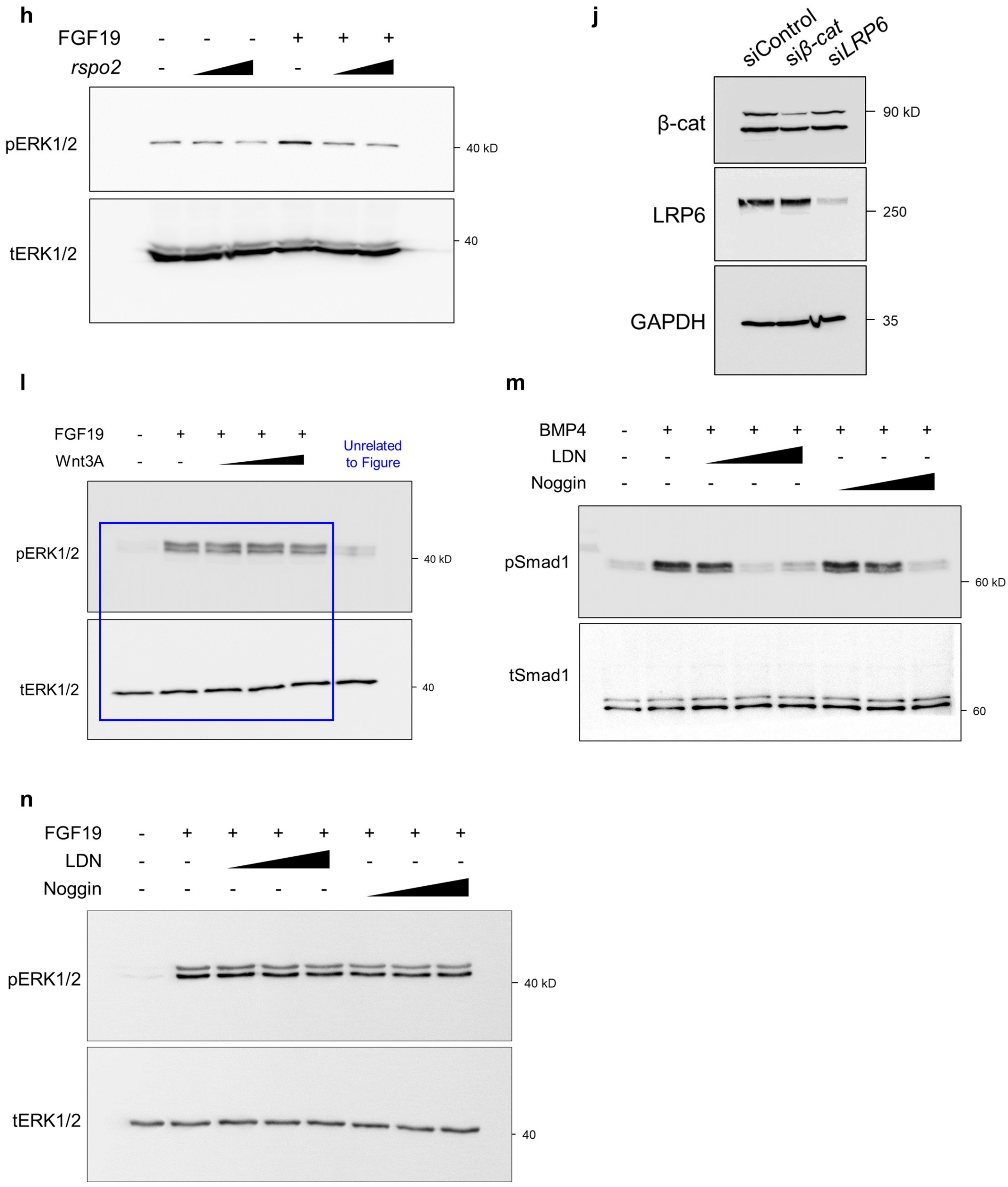

Uncropped scans of blots from Supplementary Figure 4

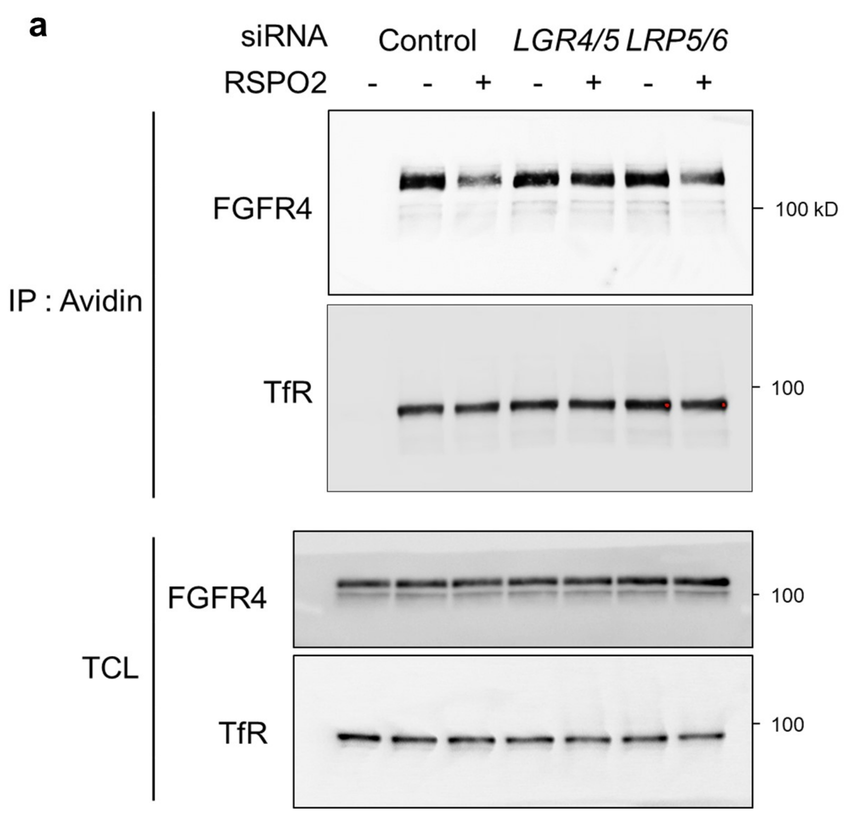

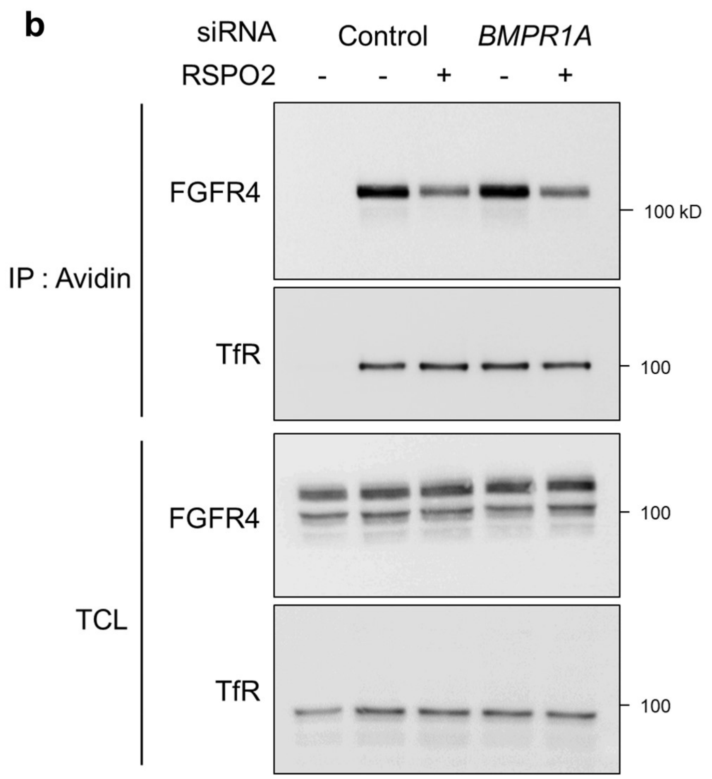

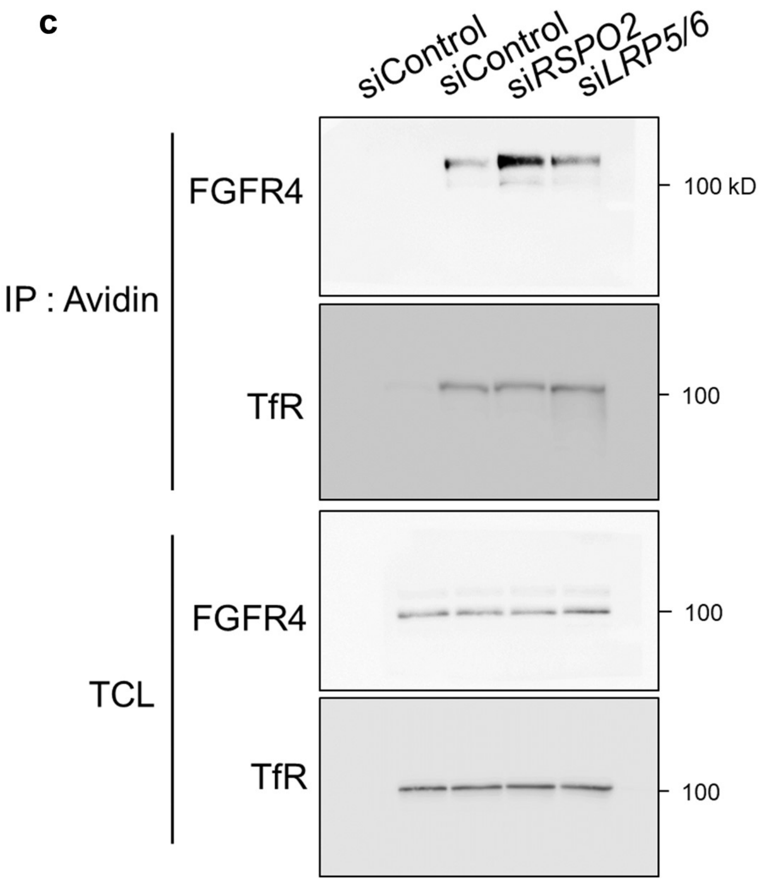

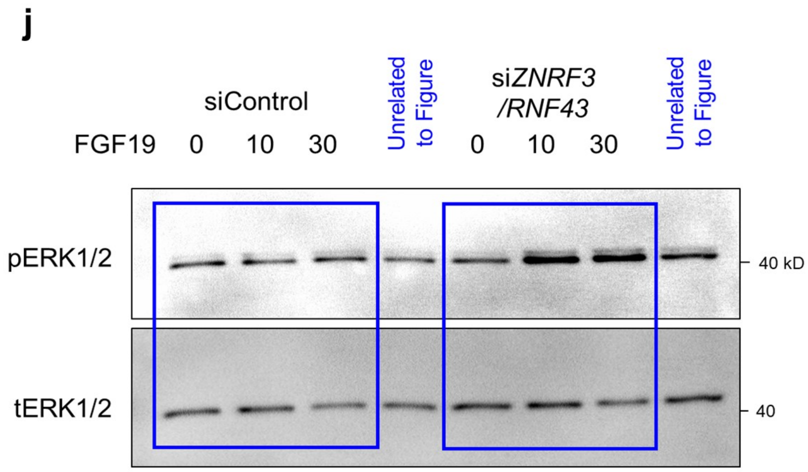

Uncropped scans of blots from Supplementary Figure 7

f

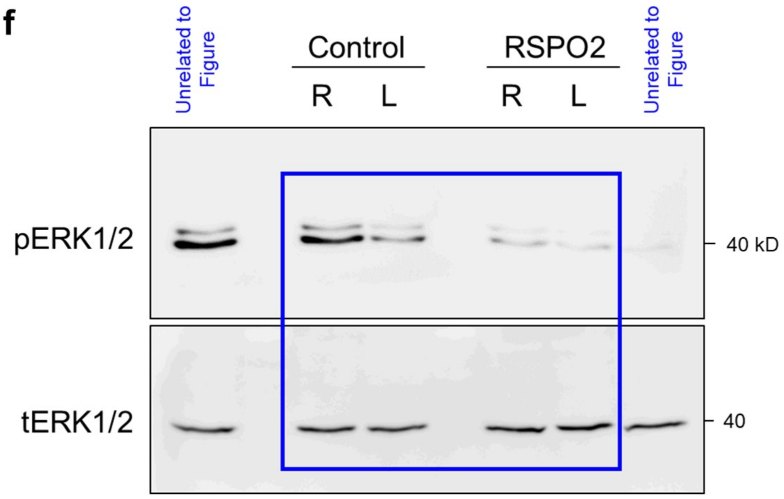

i

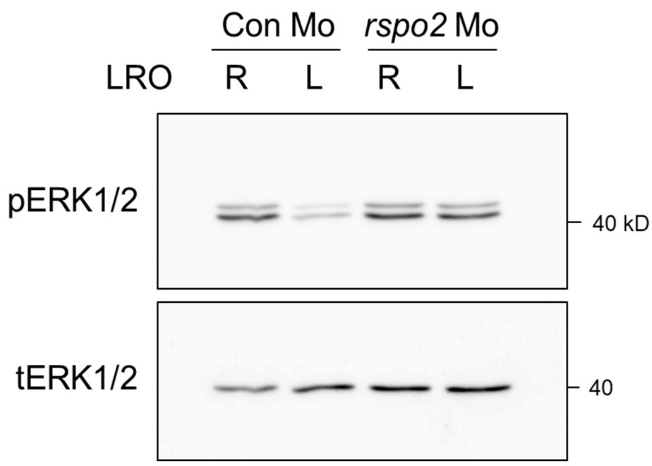

k

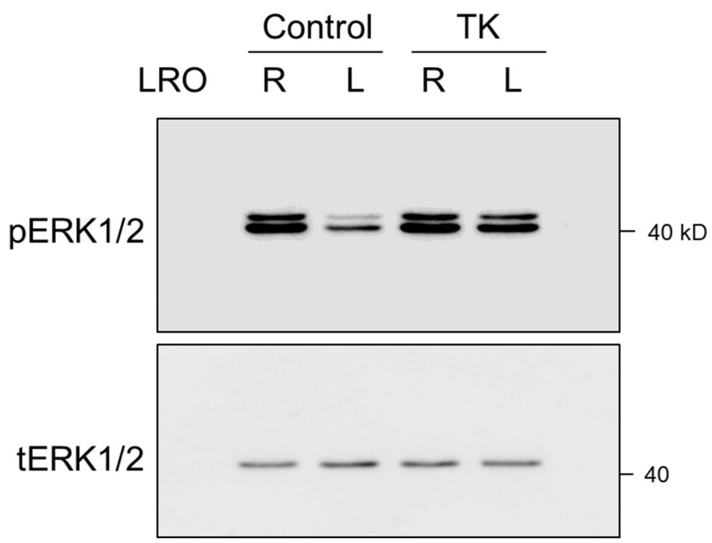

Supplement: Supplementary file 1 — Supplementary Information [file 41467_2024_44951_MOESM1_ESM.pdf]
