# Supplementary material for: R-Spondin 2 governs Xenopus left-right body axis formation by establishing an FGF signaling gradient
Source: Nat Commun. 2024 Feb 2;15:1003. doi: 10.1038/s41467-024-44951-7 (PMC10837206; doi:10.1038/s41467-024-44951-7)
Supplement: Supplementary file 3 — Reporting Summary [file 41467_2024_44951_MOESM3_ESM.pdf]

nature portfolio

Corresponding author(s): Christof Niehrs

Last updated by author(s): Dec 22, 2023

Reporting Summary

Nature Portfolio wishes to improve the reproducibility of the work that we publish. This form provides structure for consistency and transparency in reporting. For further information on Nature Portfolio policies, see our Editorial Policies and the Editorial Policy Checklist.

Statistics

For all statistical analyses, confirm that the following items are present in the figure legend, table legend, main text, or Methods section.

n/a	Confirmed	
☐	☒	The exact sample size (*n*) for each experimental group/condition, given as a discrete number and unit of measurement
☐	☒	A statement on whether measurements were taken from distinct samples or whether the same sample was measured repeatedly
☐	☒	The statistical test(s) used AND whether they are one- or two-sided<br>*Only common tests should be described solely by name; describe more complex techniques in the Methods section.*
☒	☐	A description of all covariates tested
☐	☒	A description of any assumptions or corrections, such as tests of normality and adjustment for multiple comparisons
☐	☒	A full description of the statistical parameters including central tendency (e.g. means) or other basic estimates (e.g. regression coefficient) AND variation (e.g. standard deviation) or associated estimates of uncertainty (e.g. confidence intervals)
☐	☒	For null hypothesis testing, the test statistic (e.g. *F*, *t*, *r*) with confidence intervals, effect sizes, degrees of freedom and *P* value noted<br>*Give P values as exact values whenever suitable.*
☒	☐	For Bayesian analysis, information on the choice of priors and Markov chain Monte Carlo settings
☒	☐	For hierarchical and complex designs, identification of the appropriate level for tests and full reporting of outcomes
☐	☒	Estimates of effect sizes (e.g. Cohen's *d*, Pearson's *r*), indicating how they were calculated

Our web collection on statistics for biologists contains articles on many of the points above.

Software and code

Policy information about availability of computer code

Data collection	AxioVision 40 version 4.8.2.0 was used for imaging Xenopus laevis hearts, GI tracts, phenotypes and in situ hybridizations.<br>Zeiss ZEN 2012 (black edition) version 2.5 was used for confocal microscopy imaging on LSM700.<br>Image Reader LAS 3000 version 2.21 was used to acquire western blot images.<br>Thermo Fluoroskan Ascent Software version 2.6 was used to measure luciferase activities in reporter assays and Alkaline phosphatase activities in In vitro binding assays.
Data analysis	GraphPad Prism 7 software version 7.03 was used to produce graphs and analyze statistics.<br>FIJI (ImageJ) software version 1.51k was used for confocal microscopy analyses and western blot analyses.<br>Adobe Photoshop CS6 software version 13.0 (64bit) and Microsoft PowerPoint Standard 2019 were used to edit the background in representative in situ hybridizations and Xenopus organ images.

For manuscripts utilizing custom algorithms or software that are central to the research but not yet described in published literature, software must be made available to editors and reviewers. We strongly encourage code deposition in a community repository (e.g. GitHub). See the Nature Portfolio guidelines for submitting code & software for further information.

Data

Policy information about availability of data

All manuscripts must include a data availability statement. This statement should provide the following information, where applicable:

- Accession codes, unique identifiers, or web links for publicly available datasets
- A description of any restrictions on data availability
- For clinical datasets or third party data, please ensure that the statement adheres to our policy

Website for information search:
https://www.xenbase.org/, RRID:SCR_003280
No third party datasets were analyzed in this study.
All data generated and/or analyzed during the study are available within the Figures 1-7 and Supplementary Figures 1-8.
All original data for graphs and blots are provided with this paper in the Source Data file.

Research involving human participants, their data, or biological material

Policy information about studies with human participants or human data. See also policy information about sex, gender (identity/presentation), and sexual orientation and race, ethnicity and racism.

Reporting on sex and gender	No human research participant included in the study.<br>Sex and gender of cannot be determined in Xenopus laevis embryonic stages used in the study.
Reporting on race, ethnicity, or other socially relevant groupings	No human research participant included in the study.
Population characteristics	No human research participant included in the study.
Recruitment	No human research participant included in the study.
Ethics oversight	No human research participant included in the study.

Note that full information on the approval of the study protocol must also be provided in the manuscript.

Field-specific reporting

Please select the one below that is the best fit for your research. If you are not sure, read the appropriate sections before making your selection.

[x] Life sciences [ ] Behavioural & social sciences [ ] Ecological, evolutionary & environmental sciences

For a reference copy of the document with all sections, see nature.com/documents/nr-reporting-summary-flat.pdf

Life sciences study design

All studies must disclose on these points even when the disclosure is negative.

Sample size	No sample size calculation was executed prior to the experiments. Final sample size was chosen by following Xenopus literature standards (Reference: Lee et al., Nature Communications 11, 5570 (2020), Sempou et al., Nature Communications 13, 6681 (2022)). Final sample size collected from minimum 2 independent microinjections was sufficient to perform reliable statistical analyses. All sample sizes are reported in the figures and legends.<br>For in situ hybridization with marker genes, minimum 10 embryos from 2-3 independent microinjections were analyzed.<br>For in situ hybridization to analyze expression patterns, minimum 10 embryos without microinjection were analyzed.<br>For immunohistochemistry analysis, 20 embryos were analyzed without microinjection.<br>For heart and GI tract situs anomalies, minimum 26 embryos from 2 independent microinjections were analyzed.<br>For confocal microscopy analyses with human cell lines, minimum 12 cells were analyzed from 2-3 independent experiments.<br>For confocal microscopy analyses with Xenopus tissues, minimum 3 embryos were analyzed from 2-4 independent microinjeections.<br>Independent microinjection indicates that experiments were executed on different days using different male and female Xenopus laevis.
Data exclusions	No data were excluded from the analyses.
Replication	Xenopus heart and GI tract analyses were repeated 2 times independently. Xenopus in situ hybridization were repeated 2-3 times independently. Reporter assays were repeated 2-4 times independently. Western blot analyses for FGF signaling (pERK1/2 levels and cell biotinylation assays) were repeated 2-4 times independently.<br>Western blot analyses for Wnt and BMP signaling were repeated 2 times independently. Cell surface binding assays were repeated 3-4 times independently. Confocal microscopy analyses with human cells and Xenopus explants were repeated 2-4 times independently. In vitro binding assays were repeated 2-4 times independently. We confirm that these experiments showed similar results which draw the same conclusion.<br>Exact replication numbers for each panel are indicated in the figures and corresponding legends.

Randomization	We splitted cultured cells and fertilized Xenopus eggs equally and randomly allocated to each of experimental group.
Blinding	Scoring of Xenopus phenotypes and in situ hybridization was performed without blinding by two authors (H.L, C.M.C). Since defects in Xenopus phenotypes and misexpression of in situ hybridization markers are drastically evident in microinjected embryos comparing to control embryos, the authors were already aware of the identities of analyzed samples.<br>Confocal microscopy analyses and cell surface binding assays were performed under blinding. All slides of stained culture cells and Xenopus tissues were labeled with random numbers and image acquisitions were executed under blinding.<br>During data collection (Luciferase reporter assay, AP activity measurements and western blot analyses) blinding was not executed since data were acquired in an automated manner.

Reporting for specific materials, systems and methods

We require information from authors about some types of materials, experimental systems and methods used in many studies. Here, indicate whether each material, system or method listed is relevant to your study. If you are not sure if a list item applies to your research, read the appropriate section before selecting a response.

Materials & experimental systems

n/a	Involved in the study
☐	☒ Antibodies
☐	☒ Eukaryotic cell lines
☒	☐ Palaeontology and archaeology
☐	☒ Animals and other organisms
☒	☐ Clinical data
☒	☐ Dual use research of concern
☒	☐ Plants

Methods

n/a	Involved in the study
☒	☐ ChIP-seq
☒	☐ Flow cytometry
☒	☐ MRI-based neuroimaging

Antibodies

Antibodies used	1. Rabbit anti-Phospho-ERK1/2 : Cell Signaling Technology (CST), Cat# 9101S, Lot 28 and Lot 29<br>2. Rabbit anti-ERK1/2 : Sigma, Cat# M5670, Lot 065M4813V<br>3. Rabbit anti-FGFR4 : CST, Cat# 8562S, Clone D3B12, Lot 5<br>4. Rabbit anti-Transferin receptor : CST, Cat# 13113S, Clone D7G9X, Lot 2<br>5. Mouse anti-EEA1 : BD, Cat# 610457, Clone 14/EEA1, Lot 01907<br>6. Mouse anti-Flag : Sigma, Cat# F3156, Clone M2, Lot SLBN8915V<br>7. Mouse anti-beta-Catenin : BD, Cat# 610154, Clone 14/Beta-Catenin (RUO), Lot 2300995<br>8. Rabbit anti-LRP6 : CST, Cat# 2560, Clone C5C7, Lot 11<br>9. Rabbit anti-GAPDH : CST, Cat# 2118S, Clone 14C10, Lot 10<br>10. Rabbit anti-FGFR1 : CST, Cat# 9740S, Clone D8E4, Lot 4<br>11. Mouse anti-clathrin : BD, Cat# 610499, Clone 23/Clathrin Heavy Chain (RUO), Lot 07579<br>12. Mouse anti-Lamp1 : CST, Cat# 15665S, Clone D401S, Lot 2<br>13. Rabbit anti-Phospho-Smad1: CST, Cat# 9516, Clone 41D10, Lot 9<br>14. Rabbit anti-Smad1: CST, Cat# 9743S, Lot 4<br>15. Goat anti-RSPO2: R and D systems, Cat# AF3266, Lot YBE011710A<br>16. Rat anti-HA: Roche, Cat# 11867423001, Clone 3F10<br>17. Mouse anti-active-beta-catenin: Millipore, Cat# 05-665, Clone 8E7, Lot 2654218<br>18. Sheep anti-digoxigenin-AP, Fab-Fragments: Roche, Cat# 11093274910, Lot: 16646820<br>19. Donkey anti-mouse Alexa Fluor 647 : Invitrogen, Cat# A31571, Lot 702339<br>20. Donkey anti-rat Alexa Fluor 488 : Invitrogen, Cat #A21208, Lot 2092264<br>21. Donkey anti-rabbit Alexa Fluor 546 : Invitrogen, Cat# A10036, Lot 948483<br>22. Goat anti-mouse Alexa Fluor 488 : Invitrogen, Cat# A11029, Lot 948492<br>23. Goat anti-rabbit Alexa Fluor 546 : Invitrogen, Cat# A11035, Lot 1904467<br>24. Mouse anti-Phospho-ERK1 : Santa Cruz, Cat# sc-7383, Clone E-4, Lot 1714<br>25. Goat anti-mouse IgG (H+L) HRP : Jackson ImmunoResearch, Cat# 115-035-146, Lot 137758<br>26. Goat anti-rabbit IgG (H+L) HRP : Jackson ImmunoResearch, Cat# 111-035-144, Lot 140192<br>27. Rabbit anti-ERK1/2 : GeneTex, Cat# GTX134462, Clone 2B10, Lot M-15286<br>28. Mouse anti-goat IgG (H+L) HRP : Jackson ImmunoResearch, Cat# 205-035-108, Lot 77553<br>29. Goat anti-mouse Alexa Fluor 546 : Invitrogen, Cat# A11030, Lot 1129659
Validation	1. Rabbit anti-Phospho-ERK1/2 : Tested by manufactures in HeLa, MEFs using western blotting, NIH/3T3 cells using immunofluorescent analysis. Cited more than 8000 times. Confirmed reactivity in Xenopus immunohistochemistry (Pera et al., 2015).<br>2. Rabbit anti-ERK1/2 : Tested by manufactures in K562, COS-7, HL-60, RAW-264, A431, HeLa, C2C12, JURKAT, HEK293, HEPG2, NIH-3T3, Rat brain cells using western blotting. Cited more than 180 times.<br>3. Rabbit anti-FGFR4 : Tested by manufactures in HEPG2, Huh7, COLO205 cells using western blotting. We validated with siRNA mediated knockdown in HEPG2 cells. Cited more than 40 times.<br>4. Rabbit anti-Transferin receptor : Tested by manufactures in SNB75, ZR-75, HeLa, SNB19, HT-1080 cells using western blotting.

Cited more than 46 times.
5. Mouse anti-EEA1 : Tested by manufacturers using immunofluorescent staining on human smooth muscle and western blotting on rat brain lysate.
6. Mouse anti-Flag : Commonly used and cited more than 3000 times. Tested by manufacture in immunofluorescence in canine kidney epithelial cells. Described by manufacturer as reactive to all species. We validated by western blotting using Flag-tagged RSPO2 conditioned media comparing control conditioned media.
7. Mouse anti-beta-Catenin : Commonly used and cited more than 500 times. Tested by manufacture in HeLa cells using western blotting and in A431 cells using immunofluorescent staining. We validated using siRNA mediated knockdown in HEPG2 cells.
8. Rabbit anti-LRP6 : Tested by manufactures in HepG2, HeLa, Rat2 using western blotting. Cited more than 119 times. We tested with siRNA mediated knockdown in HEPG2 cells.
9. Rabbit anti-GAPDH : Tested by manufactures in HeLa, NIH/3T3, C6, HUVEC, L929 cells using western blotting. Commonly used and cited more than 7000 times.
10. Rabbit anti-FGFR1 : Tested by manufactures in A204, KG1a, A172, HT-29 cells using western blotting. Tested by manufactures in A204, KG1, A172 cells using immunofluorescence. Cited more than 230 times.
11. Mouse anti-clathrin : Tested by manufactures in C6 cells using immunofluorescence and in SH-SY5Y, SK-N-SH cells using immunohistochemistry.
12. Mouse anti-Lamp1 : Tested by manufactures in HeLa and A172 cells by western blotting. Tested by manufactures in A-431 cells using immunofluorescence. Cited more than 60 times.
13. Rabbit anti-Phospho-Smad1: Tested by manufactures in HeLa and NIH/3t3 using western blotting. Cited more than 400 times.
14. Rabbit anti-Smad1: Tested by manufactures in HT1080, ACHN, and HUVEC cells using western blotting. Cited more than 195 times.
15. Goat anti-RSPO2: Tested by manufactures using direct ELISAs and western blotting. We validated with siRNA mediated knockdown in H1581 cells.
16. Rat anti-HA: Tested by manufactures (https://www.sigmaaldrich.com/US/en/product/roche/roahaha). We validated with BMPR1A-HA transfected H1581 cells. Cited more than 30 times.
17. Mouse anti-active-beta-catenin: Tested by manufactures using A431 cells using western blotting. We validated with Wnt3A treatment in H1581 cells and Wnt3A injection in Xenopus. Cited more than 100 times.
18. Sheep anti-digoxigenin-AP, Fab-Fragments: Validated using whole-mount in situ hybridization of zebrafish embryos (Carlisle et al., 2013). Commonly used for In situ hybridization of Xenopus embryos. Cited more than 750 times.
19. Donkey anti-mouse Alexa Fluor 647 : Tested by manufacture using immunofluorescence on HeLa cells. Commonly used and cited more than 1700 times.
20. Donkey anti-rat Alexa Fluor 488 : Tested by manufacture using immunofluorescence on A549 cells. Commonly used and cited more than 1300 times.
21. Donkey anti-rabbit Alexa Fluor 546 : Tested by manufacture using immunofluorescence on HeLa cells. Commonly used and cited more than 400 times.
22. Goat anti-mouse Alexa Fluor 488 : Tested by manufacture using immunofluorescence on HeLa, HDFn and BPAE cells. Commonly used and cited more than 5000 times.
23. Goat anti-rabbit Alexa Fluor 546 : Tested by manufacture using immunofluorescence on HeLa and HCT116 cells. Commonly used and cited more than 1000 times.
24. Mouse anti-Phospho-ERK : Tested by manufacture using western blotting on HeLa, A-431, SK-MEL-24, Jurkat, K-562 and NIH/3T3 cells. Confirmed reactivity in Xenopus immunofluorescence (Kinoshita et al., 2020).
25. Goat anti-mouse IgG (H+L) HRP : Commonly used and cited more than 600 times.
26. Goat anti-rabbit IgG (H+L) HRP : Commonly used and cited more than 6000 times.
27. Rabbit anti-ERK1/2 : Tested by manufacture using western blotting on MDCK, PG-4, Neuro2A, NIH-3T3, Raw264.7 and C2C12 cells. Cited more than 15 times.
28. Mouse anti-goat IgG (H+L) HRP : Cited 30 times. We validated in H1581 cells with western blotting using goat anti-RSPO2 antibody.
29. Goat anti-mouse Alexa Fluor 546 : Tested by manufacture using immunofluorescence on HeLa, 293F, and stromal cells. Commonly used and cited more than 800 times.

Eukaryotic cell lines

Policy information about cell lines and Sex and Gender in Research

Cell line source(s)

HEK293T (ATCC) : Human, female; HEPG2 (ATCC) : Human, male; H1581 (ATCC; Gift from Dr. R. Thomas) : Human, male; L cells (ATCC) : Mouse, male; L Wnt-3A cells (ATCC) : Mouse, male

Authentication

None of the cell lines were authenticated.

Mycoplasma contamination

All the cells were tested and negative for mycoplasma contamination.

Commonly misidentified lines
(See ICLAC register)

No commonly misidentified lines used in this study.

Animals and other research organisms

Policy information about studies involving animals; ARRIVE guidelines recommended for reporting animal research, and Sex and Gender in Research

Laboratory animals

Adult Xenopus laevis frogs were obtained from the National Xenopus Resource (NXR), the European Xenopus Resource Centre (EXRC) and Nasco. Adults female Xenopus laevis (> 10 cm) were used to obtain eggs. Adults male Xenopus laevis (> 6 cm) were used to

obtain testis for in vitro fertilization. All animal experiments were approved by the state review board of Baden-Württemberg, Regierungspräsidium Karlsruhe, Germany (permit number 35-9185.81/G-141/18, G-116/23).

Xenopus laevis embryos were injected with Morpholino, mRNA, DNA, recombinant proteins, or small molecule inhibitors at St. 2-3 or St. 15. Embryos at St. 18-19 or St. 26-31 were analyzed for In situ hybridization. Embryos at St. 14 or St. 18 were analyzed for immunofluorescent staining. Embryos at St. 42 were analyzed for heart and gut situs. Animal caps were explanted at St. 8-9 and analyzed at St. 13 for western blotting and at St. 9 for immunofluorescence. Embryos at St. 18-19 were analyzed for western blotting. All stages are indicated in the corresponding figures and legends.

Wild animals	The study did not involve wild animals.
Reporting on sex	Sex of the Xenopus embryos is not distinguishable at these stages and unrelevant to the study, therefore not determined.
Field-collected samples	The study did not involve field-collected samples.
Ethics oversight	All Xenopus laevis experiments were approved by the state review board of Baden-Württemberg, Germany (permit number 35-9185.81/G-141/18 and G-116/23 (Regierungspräsidium Karlsruhe)) and performed according to the federal and institutional regulations.

Note that full information on the approval of the study protocol must also be provided in the manuscript.
